# Psychometric properties of the Chinese version of the Exercise Self-Efficacy Scale for the Transtheoretical Model: a confirmatory analysis among Chinese children and adolescents

**DOI:** 10.1186/s12889-023-17596-2

**Published:** 2024-01-10

**Authors:** Liying Yao, Ke Zhou, Yanli Zhou, Yee Cheng Kueh, Hongyou Liu, Zhongbiao Liu, Mingzhu Pan, Garry Kuan

**Affiliations:** 1 School of Physical Education, Shangrao Normal University, Shangrao, Jiangxi 334000 China; 2 Exercise and Sports Science Programme, School of Health Sciences, Universiti Sains Malaysia, Kubang Kerian, Kelantan 16150 Malaysia; 3 Sports Reform and Development Research Center of Henan University, School of Physical Education, Henan University, Kaifeng, China; 4 Biostatistics and Research Methodology Unit, School of Medical Sciences, Universiti Sains Malaysia, Kubang Kerian, Kelantan Malaysia; 5 School of Physical Education & Sports Science, South China Normal University, Guangzhou, China

**Keywords:** Validity, Reliability, Physical activity, Forward-backward translation

## Abstract

**Background:**

Self-efficacy has been recognized as a critical component in people’s participation and maintenance of physical activity. This study aims to validate the Chinese version of the Exercise Self-Efficacy Scale (ESE) among Chinese children and adolescents using confirmatory factor analysis (CFA).

**Methods:**

A cross-sectional study was conducted on two primary and two secondary schools in central China. The ESE scale was translated into Chinese (ESE-C) using the standard forward-backward translation method. Data were analyzed using Mplus 8 for the CFA.

**Results:**

The final model showed a satisfactory level of goodness-of-fit (CFI = 0.918; TLI = 0.905; SRMR = 0.043; RMSEA = 0.066), indicating a good construct validity of the ESE-C for children and adolescents in mainland China. Furthermore, the final ESE-C model achieved composite reliability values of 0.963 and average variance extraction values of 0.597, indicating sufficient convergent and discriminant validity. Besides, the Cronbach’s alpha value was 0.964, demonstrating excellent internal consistency of the ESE-C scale.

**Conclusion:**

The ESE-C scale is a valid instrument for assessing exercise self-efficacy among children and adolescents in mainland China.

## Introduction

Insufficient physical activity (PA) and sedentary behavior (SB) have emerged as a global epidemic among children and adolescents, displaying a troubling upward trend [1, 2]. This concerning situation is caused by various factors and poses potential risks to children’s future health. Given the crucial role of PA in children’s growth, development, and mental well-being [3–5], there is an urgent need for effective interventions to enhance their PA levels.

China is no exception to this trend. After experiencing dramatic economic growth and improved living conditions, the levels of PA among children and adolescents have also raised concerns [6]. According to WHO guidelines, more than 87% of Chinese children and adolescents do not meet the recommended PA recommendations [2]. However, research on PA and SB in Chinese populations remains limited, with existing studies highlighting significant concerns across all age groups, especially among children and adolescents aged 8 to 12 [7–10].

To promote and sustain youth participation in PA, a promising approach is to implement the Transtheoretical Model (TTM), which is commonly used in mental health counseling to describe behavioral change [11–12]. TTM has found extensive application in various health-related domains, with a particular emphasis on promoting engagement in PA and ensuring adherence to exercise routines [13]. TTM perceives behavior change as a dynamic process, encompassing cognitive, affective, and behavioral strategies while delineating individuals’ readiness for change and progression through distinct stages [14]. Notably, a substantial body of research has utilized TTM to develop interventions aimed at increasing PA participation [13, 15–17]. The fundamental constructs within TTM include stages of change, change processes, decision balance, self-efficacy, and temptation [16]. Our study’s primary focus was on evaluating the Chinese version of the TTM exercise self-efficacy scale among Chinese children and adolescents.

Within the TTM framework, self-efficacy holds significant importance as a psychological construct. Self-efficacy (SE) refers to a person’s belief in their ability to overcome obstacles and successfully complete tasks that lead to positive outcomes [18]. It has been found that individuals with high levels of self-efficacy tend to experience more positive emotions and successfully accomplish their tasks [18]. Importantly, physical self-efficacy is considered a reliable predictor of an individual’s engagement and adherence to physical activity [19]. Additionally, SE is a major predictor of the type of PA one chooses, the level of effort invested in the chosen activity, and how one deals with obstacles during the activity [20].

To assess exercise self-efficacy, researchers have developed several scales, including the 18-item Exercise Self-Efficacy Scale (ESE) by Bandura [21], the 9-item Self-Efficacy for Exercise Scale (SEE) by Resnick and Jenkins [22], the 17-item Physical Activity Self-Efficacy Scale (PASES) by Saunders et al. [23], the 16-item Cardiac Exercise Self-Efficacy Scale (CESEI) by Hickey et al. [24], the 10-item SCI Exercise Self-Efficacy Scale (ESES) by Kroll et al. [25], and the 14-item Tai Chi Exercise Self-Efficacy Scale (TCSE) by Li et al. [26]. All six scales have undergone psychometric testing, but the last three are specifically targeted to particular populations. The CESEI consists of 16 items measuring an individual with cardiovascular risks’ confidence to engage in physical activity in various situations, each rated on a 5-point Likert scale from 1 = “very unconfident” to 5 = “quite a lot of confidence” [24]. The ESES was designed to assess the self-confidence of participants with spinal cord injuries in performing regular physical activities and exercise on a 4-point Likert scale (ranging from 1 = not at all true to 4 = always true) [25]. The TCSE was designed to assess participants’ perceived self-efficacy in performing Tai Chi, with responses to each item ranging from 0 (not at all confident) to 100 (very confident) [26].

Among the first three scales, the nine-item SEE developed by Resnick and Jenkins was designed to assess people’s confidence to continue exercising in the face of barriers to exercise, with items rated from 1 (not confident) to 10 (very confident). Its brevity makes it a time-efficient and practical choice for large-scale studies, especially in busy clinical settings or when used alongside multiple measures [22]. The Chinese version of the SEE scale has been validated among older adults in Taiwan and middle-aged patients with coronary heart disease in Hong Kong [27, 28]. The PASES, developed by Saunders et al., uses a 5-point Likert scale containing 17 indicators divided into 3 factors. However, some subsequent studies do not support this multidimensional scale. Therefore, some researchers generated a shortened 8-item unidimensional model (S-PASES), which has received more supportive research.

However, the Exercise Self-Efficacy Scale (ESE) developed by Bandura [21] currently stands as the most widely used and efficient tool for assessing an individual’s exercise self-efficacy. With 18 items, the ESE offers a more comprehensive evaluation, encompassing a broader range of exercise-related situations and challenges [29]. It has been extensively validated in various populations [19, 29–36], demonstrating reliability and validity across diverse cultural contexts. Additionally, the ESE is based on Bandura’s ‘Social Cognitive Theory,’ a widely accepted and influential psychological framework, further supporting its theoretical foundations.

Nevertheless, it is important to note that no validated Chinese version of the ESE scale is currently available [37]. As a result, researchers in mainland China resort to self-developed or differently translated versions of the ESE scales [38–43]. This discrepancy poses a challenge in effectively applying the Transtheoretical Model (TTM) to enhance PA levels across different groups in China. Therefore, our investigation focuses on assessing the validity and reliability of the Chinese version of the Exercise Self-Efficacy Scale (ESE-C) specifically among children and adolescents in China. The unique characteristics of this age group, including their differing comprehension skills compared to adults, warrant a thorough evaluation of the scale’s suitability in this context. As a result, our study serves as a crucial prerequisite, ensuring that the ESE-C is a valid and reliable tool for measuring exercise self-efficacy in children and adolescents, thus enabling more effective research in this area in the future.

## Materials and methods

### Participants

A total of 1,573 primary and secondary school students from Jiangxi, China, willingly participated in this study, comprising 894 boys and 679 girls, with a mean age of 12 years (SD = 1.68). Among them, 842 (53.5%) were primary school students, and 731 (46.5%) were secondary school students. The majority of participants identified as Han Chinese (99.2%). Prior to their involvement in the study, informed consent from parent or legal guardian were obtained from all participants.

### Measures

The study included two self-reported scales. A demographic survey collected information on participants’ age, gender, grade, and ethnic background. The Chinese version of the ESE scale (ESE-C), based on Bandura’s original version [18], was used. It comprised 18 items measuring self-efficacy, represented by a single factor. Participants rated themselves on a 5-point Likert scale, ranging from 1 (Not at all confident) to 5 (Completely confident).

### Ethics and procedures

Prior to data collection, ethical approval was obtained from both the USM Human Research Ethics Committee (USM/JEPeM/21,090,638) and the Jiangxi Medical College Human Research Ethics Committee (Approval No: (RH)2022-5). The translation process of the ESE scale into Chinese followed Brislin’s standard forward-backward translation procedure (1986) [44].

Two bilingual researchers first independently translated the scale, collaboratively forming a draft version through comparison and discussion. This draft underwent evaluation by five experts in sports science, sports psychology, and physical education, who provided valuable insights and refinements. For linguistic precision and cross-cultural adaptability, two additional bilingual researchers back-translated the improved Chinese version into English, iteratively modifying it until achieving consensus with the original scale. Furthermore, ten native Chinese speakers reviewed the draft scale, identifying and addressing any ambiguities or difficulties, playing a pivotal role in shaping the final version. Subsequently, a pilot study involved ten native Chinese students answering the scale, revealing no new concerns about content and confirming the scale’s finalization.

Following the translation process, a convenience sampling method was employed for participant selection, enrolling 1573 children and adolescents aged 9–15 years. The sampling involved the deliberate selection of four public or private primary and middle schools in Shangrao City, Jiangxi province. Shangrao City was stratified into urban and suburban areas, and two primary and two middle schools were conveniently recruited from each zone. To ensure a representative sample, five classes were then randomly chosen from each of the four schools, covering grades 4 through 9. Data collection took place on the school campuses. Prior to questionnaire administration, the researcher briefly introduced it to the students and distributed the informed consent form to students and their parents or legal guardians. Participants were requested to answer the questions honestly and to the best of their ability.

### Statistical analysis

The validity of the hypothesized ESE-C measurement models was assessed using confirmatory factor analysis (CFA) with the statistical software Mplus 8. In this analysis, all 18 items were treated as observed variables, while the factor was considered the latent variable.

To ensure the reliability of our analysis, we checked the data for multivariate normality. The results indicated that the assumption of normality was not met, as evidenced by significant outcomes in the Mardia multivariate skew (*p* <.001) and kurtosis (*p* <.001) tests. As a result, to address the non-normality in subsequent CFAs, we opted for the robust maximum likelihood estimator (MLR), which is well-suited for handling such data [45, 46].

To establish the validity of the questionnaire, it is customary among researchers to present multiple fit indices [47]. Accordingly, we utilized several fit indices along with their respective threshold values: comparative fit index (CFI) and Tucker and Lewis index (TLI) with desired values above 0.90, root mean square error of approximation (RMSEA) with a desired value below 0.08, probability RMSEA with a desired value above 0.07, and standardized root mean square residual (SRMR) with a desired value below 0.08 [48].

Items with factor loadings below 0.4 were identified as problematic [49, 50] and underwent evaluation for potential removal after conducting a thorough theoretical review. During the process of model re-specification, we examined the CFA modification index (MI) to identify opportunities for refining the measurement models and achieving the best fit. The re-specification process involved introducing correlations between items’ residuals within the same factor, which was supported by sound theoretical reasoning [49].

To assess the reliability of the final ESE-C measurement model, we used composite reliability (CR) based on Raykov’s method in Mplus [51], CR is similar to Cronbach’s Alpha, indicating internal consistency, and we set a minimum acceptable threshold of 0.60 and above [52] for reliability. Convergent validity was evaluated using Average Variance Extracted (AVE), with values above 0.5 indicating strong convergent validity [53]. Additionally, McDonald’s Omega was calculated to provide a complementary perspective on reliability, addressing unidimensionality and measurement errors more robustly than Cronbach’s alpha [54]. Reporting both coefficients enhances the comprehensiveness of our reliability assessment, contributing to a nuanced understanding of the scale’s internal consistency and reinforcing the robustness of our findings. These evaluations rigorously confirm the reliability and convergent validity of the ESE-C measurement model [53].

To assess discriminant validity, inter-factor correlations were examined. Adequate discriminant validity is established when factor correlations remain less than or equal to 0.85 [55]. Test-retest reliability was evaluated using a sub-sample of 76 participants, involving the calculation of the Intraclass Correlation Coefficient (ICC). Reliability was considered satisfactory when ICC values exceeded 0.70 [56]. This approach confirms the distinctiveness of the factors and ensures the stability and consistency of measurements over time.

## Results

### Descriptive statistics

A total of 1573 students, aged between 9 and 15 years old (M = 12), participated in the study (as shown in Table 1). The participants comprised 842 primary school students and 731 secondary school students. Among them, 894 were boys, and 679 were girls. The majority of participants belonged to the Han Chinese ethnic group, consistent with the demographic composition of most regions in China.

**Table 1:** Descriptive statistics of demographic characteristics and study variables of participants (*n* = 1573)

Characteristics	Frequency	Percentage (%)	Mean (SD)
**Gender**			
Boys	894	56.8%	
Girls	679	43.2%	
**Age (year)**			12 (1.68)
9	61	3.9%	
10	217	13.8%	
11	300	19.1%	
12	291	18.5%	
13	285	18.1%	
14	246	15.6%	
15	173	11.0%	
**Ethnicity**			
Han Chinses	1561	99.2%	
Ethnic Minorities	12	0.8%	
**Education Level**			
Primary school	842	53.5%	
Secondary school	731	46.5%	

*Notes*: Ethnic Minorities include Mongolian, Zhuang, She, Miao, Hui, Li, Yao, and Tujia

### Confirmatory factor analysis

**Fig. 1:**
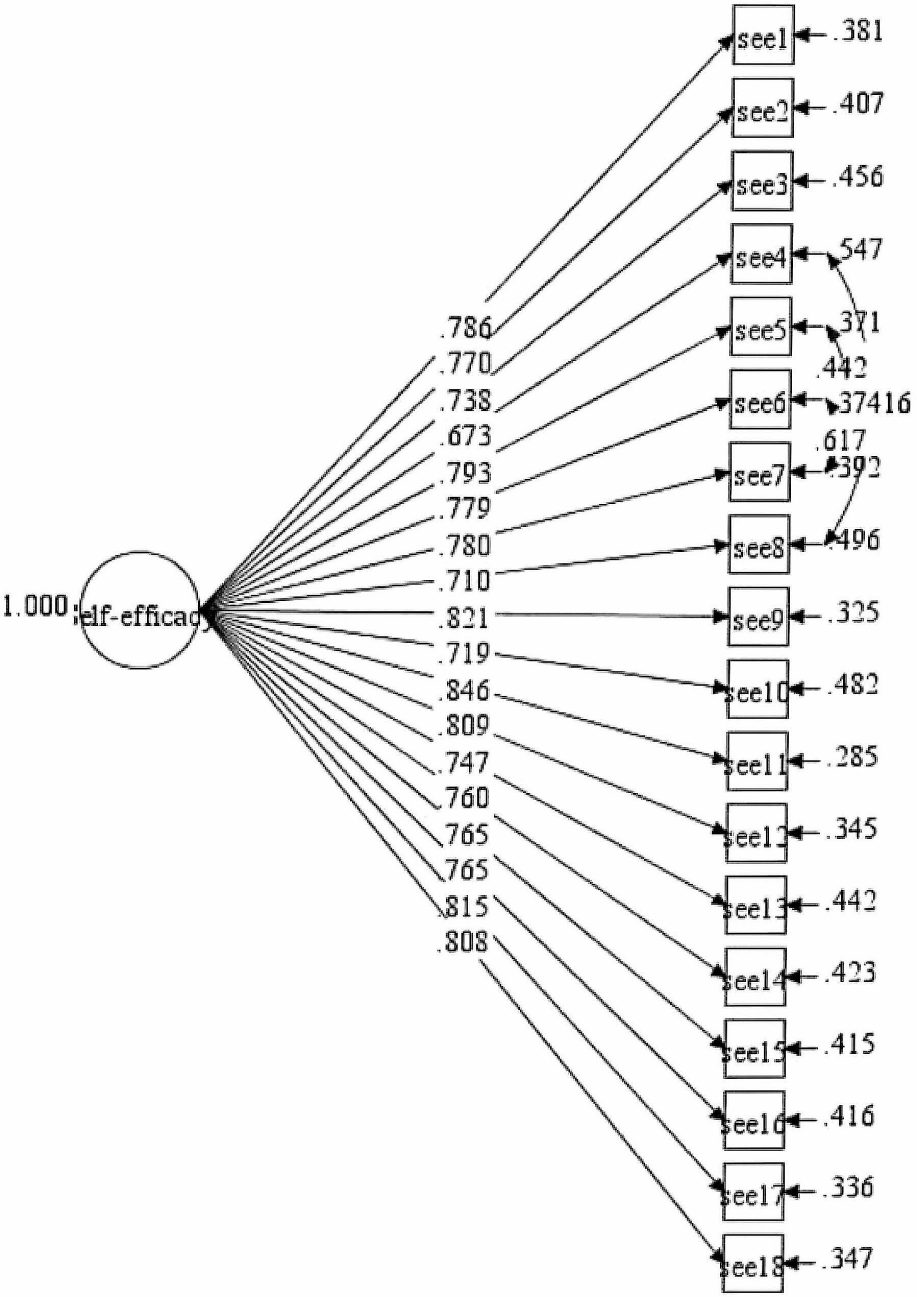
Modified ESE-C measurement model (Model-1, all samples)

The tests of the initial hypothesis ESE-C model (model-1) exhibited poor overall fit indices except for SRMR (CFI = 0.869; TLI = 0.852; SRMR = 0.050; RMSEA (CI: 90%) = 0.082 (0.079, 0.086)). To improve the fit index of the model, we iteratively added several correlations between items’ residuals in the model, starting with the highest value of the modification index (MI) and gradually addressing lower MI values until the model adequately fits the data. The correlations were added on items’ residuals within the same factor. Through this re-specification process, we arrived at the final model (see Table 2, Final model, see Fig. 1). The final model demonstrated a satisfactory level of goodness-of-fit with fit indices: CFI = 0.918; TLI = 0.905; SRMR = 0.043; RMSEA (CI: 90%) = 0.066(0.062, 0.070), indicating a well-validated ESE-C for our study with children and adolescents in mainland China.

**Table 2:** Summary for ESE-C constructs model fit indices (Model-1)

Path Model	RMSEA (90%CI)	CFI	TLI	SRMR
Initial ESE-C	0.082(0.079, 0.086)	0.869	0.852	0.050
Final ESE-C	0.066(0.062,0.070)	0.918	0.905	0.043

*Notes*: Four correlations on items’ residuals were added: ESE7 with ESE6, ESE7 with ESE5, ESE6 with ESE5, and ESE8 with ESE4.

Furthermore, our study included children and adolescents from both primary and secondary schools. To determine the replicability of the Chinese version of the ESE scale among students at different academic levels, we divided the participants into a primary school group (Model-2) and a secondary school group (Model-3) based on the overall sample (Model-1) and tested the models’ fitness individually.

**Fig. 2:**
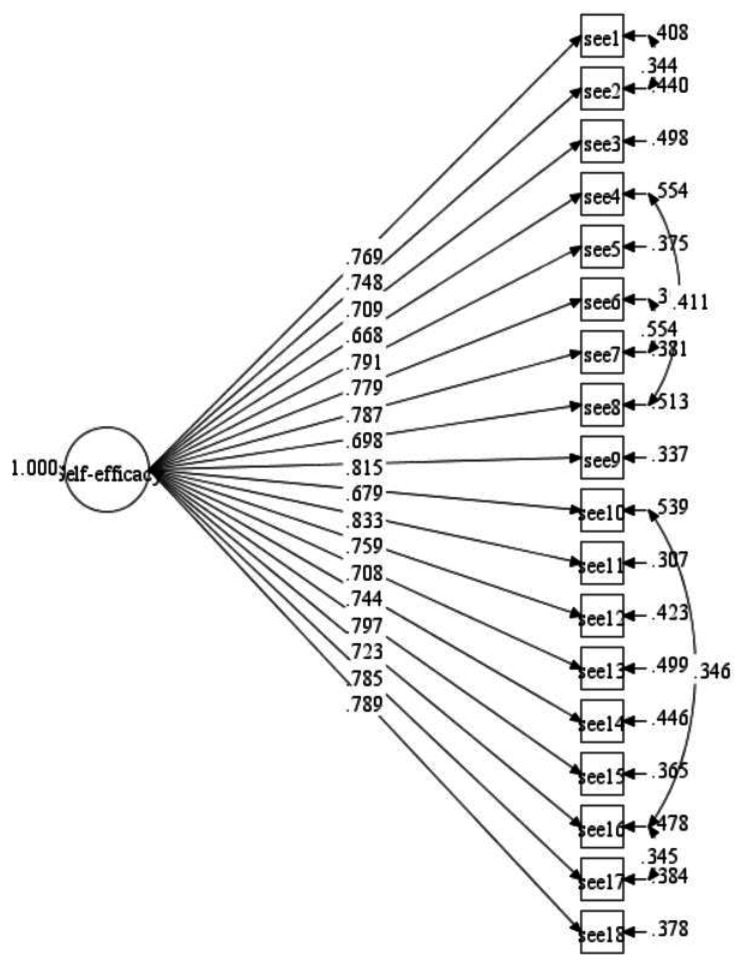
Modified ESE-C measurement model (Model-2, primary school students)

The model fit indices for Model-2 (primary school) are presented in Table 3. The initial hypothesis model (Model-2) also exhibited poor overall fit indices: CFI = 0.861, TLI = 0.843, SRMR = 0.055, RMSEA (CI: 90%) = 0.082 (0.077, 0.087). To improve the model’s fit, we added five correlations between items’ residuals to the initial ESE-C Model-2 (ESE7 with ESE6; ESE2 with ESE1; ESE8 with ESE4; ESE16 with ESE10; ESE17 with ESE16). All the correlations were added on items’ residuals within the same factor. The final model fit indices then satisfied the required threshold with CFI = 0.923, TLI = 0.909, SRMR = 0.047, RMSEA (CI: 90%) = 0.062 (0.057, 0.067) (see Table 3, Final Model-2, see Fig. 2).

**Table 3:** Summary for ESE-C constructs model fit indices (Model-2)

Path Model	RMSEA (90%CI)	CFI	TLI	SRMR
Initial Model-2	0.082(0.077, 0.087)	0.861	0.843	0.055
Final Model-2	0.062(0.057,0.067)	0.923	0.909	0.047

*Notes*: five correlations on item’s residuals were ESE7 with ESE6; ESE2 with ESE1; ESE8 with ESE4; ESE16 with ESE10; ESE17 with ESE16.

**Fig. 3:**
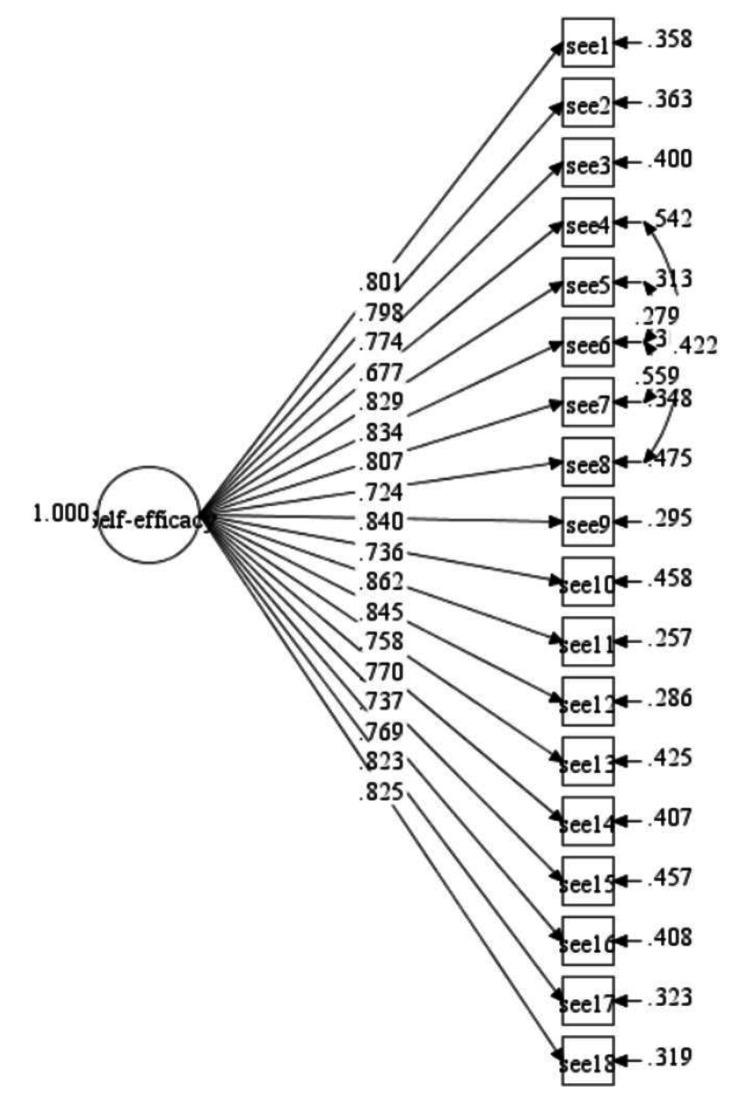
Modified ESE-C measurement model (Model-3, secondary school students)

The initial model fit indices of Model-3 (Secondary school) also showed poor overall fit indices with CFI = 0.875; TLI = 0.858; SRMR = 0.047; RMSEA (CI: 90%) = 0.086(0.080, 0.091). To enhance the model’s fit, we added three correlations between items’ residuals to the initial ESE-C Model-3 (ESE7 with ESE6; ESE6 with ESE5; ESE8 with ESE4). Then, the final model fit indices met the threshold requirement with CFI = 0.917; TLI = 0.904; SRMR = 0.042; RMSEA (CI: 90%) = 0.071(0.065, 0.076). (see Table 4, Final Model-3, see Fig. 3).

**Table 4:** Summary for ESE-C constructs model fit indices (Model-3)

Path Model	RMSEA (90%CI)	CFI	TLI	SRMR
Initial Model-3	0.086(0.080, 0.091)	0.875	0.858	0.047
Final Model-3	0.071(0.065,0.076)	0.917	0.904	0.042

*Notes*: three correlations on item’s residuals were SEE7 with SEE6; SEE6 with SEE5; SEE8 with SEE4.

### Composite reliability (CR) and discriminant validity

As shown in Table 5, the final ESE-C model achieved CR values of 0.963 and AVE values of 0.597, indicating sufficient convergent and discriminant validity. Model-2 (Primary school) and Model-3 (Secondary school) also yielded acceptable CR and AVE values. Therefore, the model achieved adequate convergence and discriminant validity.

**Table 5:** Summary of CR, AVE value for each model

Variables	CR	AVE
Model-1 (All sample)	0.963	0.597
Model-2 (Primary school)	0.960	0.571
Model-3 (Secondary school)	0.956	0.550

### Internal consistency

The Cronbach alpha values for Model-1, Model-2 (Primary school), and Model-3 (Secondary school) were 0.964, 0.961, and 0.968, respectively (see Table 6). Likewise, the McDonald’s Omega values for these models were identical, indicating excellent internal consistency across all three.

**Table 6:** Summary of Cronbach alpha and McDonald’s Omega value for each model

Variables	Cronbach alpha	McDonald’s Omega
Model-1 (All sample)	0.964	0.964
Model-2 (Primary school)	0.961	0.961
Model-3 (Secondary school)	0.968	0.968

### Test-retest reliability

A test-retest reliability analysis spanning two weeks and involving 76 participants was conducted to assess the stability of responses. The mean score on the ESE-C scale exhibited a slight shift from 54.5 (Day 1, SD = 14.5) to 55.3 (Day 14, SD = 14.6). The ICC value was 0.870 (95% CI = 0.815, 0.921, *p*-value < 0.001), indicating adequate stability.

## Discussion

This study aimed to assess the reliability and validity of the Chinese version of the Exercise Self-Efficacy Scale (ESE-C) using Confirmatory Factor Analysis (CFA) among Chinese children and adolescents. The findings revealed that no problematic items were identified in the ESE-C, leading to the retention of all 18 items in the final model, which demonstrated good reliability.

The significance of physical activity (PA) for individuals of all ages has gained widespread recognition [51]. To encourage and sustain youth involvement in PA, the adoption of the Transtheoretical Model (TTM) has emerged as a promising strategy in various contexts [14, 15, 17]. TTM incorporates behavioral and cognitive strategies, placing emphasis on exercise persistence and its interplay with psychological factors [12]. Self-efficacy, a fundamental construct of TTM, plays a central role in motivating individuals to initiate and maintain PA [21]. To assess individuals’ exercise self-efficacy, various measurement tools have been developed, with Bandura’s Exercise Self-Efficacy scale being widely acknowledged [21].

However, the application of the Transtheoretical Model (TTM) and the Exercise Self-Efficacy Scale (ESE) to promote PA participation among diverse populations in mainland China remains limited. Existing studies also lack comprehensive validation of the Chinese version of the ESE scale (ESE-C), leading to wide variations in the questionnaires used by scholars in mainland China when applying the TTM and the ESE scale [36–38, 42, 43, 57–61]. Some researchers utilized self-developed scales [58], while others employed different versions of the ESE scale, such as Saunders’ 17-item Physical Activity Self-Efficacy Scale (PASES) [26] or Resnick and Jenkins’ 9-item Exercise Self-Efficacy Scale (ESES) [22, 61]. Notably, there has been no validation study of Bandura’s original Exercise Self-Efficacy Scale (ESE) in Chinese yet. This lack of thorough validation poses a hindrance to further research in mainland China. Therefore, this study sought to validate the reliability and validity of the ESE-C among Chinese children and adolescents, confirming its suitability for this age group.

While this study makes a significant contribution to the existing literature by translating and validating the ESE scale in Chinese, supporting the validity of the ESE-C, it is crucial to acknowledge certain limitations. The study’s results are specific to Chinese children and adolescents, with the sampling area confined to one city in southern China. As a result, the generalizability of the findings to other age groups and educational levels remains uncertain. Future research should aim to replicate this study in diverse regions of China, encompassing various age groups and educational backgrounds, to further validate the applicability of the ESE-C.

Furthermore, despite its valuable contributions, this study lacks a cross-cultural comparison with other cultures. To address this limitation, we recommend that future studies undertake comparisons of the CFA model of ESE across different study populations or countries. Techniques such as multigroup analysis and invariance tests could be employed for a comprehensive cross-cultural understanding.

Additionally, this study employed a non-probability sampling method to recruit participants, which may limit its representativeness for the entire Chinese population. We highly recommend implementing improved sampling methods in future research to enhance the scope and representativeness of data collection. It would be advantageous to consider diverse socio-economic backgrounds, different age strata, various educational backgrounds, and both urban and rural areas when conducting studies. By doing so, the findings will be more applicable and generalizable to the entire population of Chinese children and adolescents. A broader and more inclusive approach to data collection will contribute to a more comprehensive understanding of exercise self-efficacy among young individuals in China and facilitate the development of effective interventions to promote physical activity and well-being in this population.

## Conclusion

This study provides strong support for the validity and reliability of the Exercise Self-Efficacy Scale among Chinese children and adolescents. Consequently, the scale can serve as a valuable tool for measuring exercise self-efficacy in this specific age group.

## Data Availability

The dataset used during the current study is available on reasonable request from LY and GK.
